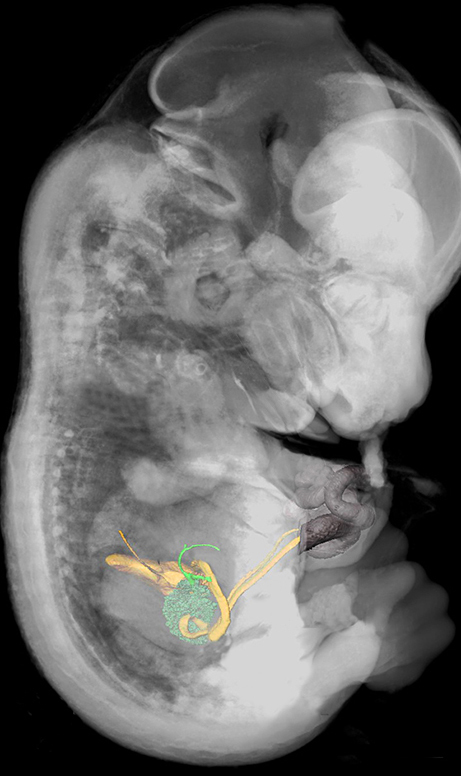# Advancing congenital defect phenotyping using HREM imaging

**Published:** 2014-10

Knockout mouse lines that show embryonic and perinatal lethality are valuable models to investigate the genetic pathways that are associated with human congenital diseases. To elucidate the relationship between a specific gene and a developmental defect, detailed screening of knockout phenotypes is pivotal. Here, Wolfgang Weninger and colleagues used high-resolution episcopic microscopy (HREM) to screen the morphological phenotypes of embryonic day 14.5 (E14.5) embryos of 34 mouse strains that produce prenatally lethal offspring. The authors developed a reliable and ergonomic screening protocol to efficiently and comprehensively score structural abnormalities in those embryos. Their approach enabled them to detect a total of 58 defects that might be missed by employing alternative three-dimensional imaging methods and scoring systems. Many of these defects might be causal to embryonic or perinatal mortality. The results demonstrate that HREM combined with a systematic screening protocol enables more efficient phenotyping of E14.5 mouse embryos than any alternative approach. Such a method will contribute to advancing our knowledge of normal tissue and organ development, and of the causality of congenital diseases. **Page 1143**

**Figure:**